# Crosstalk between B cells and neutrophils in rheumatoid arthritis

**DOI:** 10.1111/imm.13412

**Published:** 2021-09-08

**Authors:** Utsa Karmakar, Sonja Vermeren

**Affiliations:** ^1^ Centre for Inflammation Research Institute for Regeneration and Repair The University of Edinburgh Edinburgh UK

**Keywords:** anti‐citrullinated protein antibodies, B cells, citrullination, dysbiosis, immune complexes, neutrophil extracellular traps, neutrophils, peptidylarginine deiminase, rheumatoid arthritis

## Abstract

Rheumatoid arthritis (RA) is a chronic, systemic autoimmune disease without known cure that primarily affects synovial joints. RA has a prevalence of approximately 1% of the population worldwide. A vicious circle between two critical immune cell types, B cells and neutrophils, develops and promotes disease. Pathogenic anti‐citrullinated protein antibodies (ACPA) directed against a range of citrullinated epitopes are abundant in both plasma and synovial fluid of RA patients. In addition to stimulating numerous cell types, ACPA and other autoantibodies, notably rheumatoid factor, form immune complexes (ICs) that potently activate neutrophils. Attracted to the synovium by abundant chemokines, neutrophils are locally stimulated by ICs. They generate cytokines and release cytotoxic compounds including neutrophil extracellular traps (NETs), strands of decondensed chromatin decorated with citrullinated histones and granule‐derived neutrophil proteins, which are particularly abundant in the synovial fluid. In this way, neutrophils generate citrullinated epitopes and release peptidylarginine deiminase (PAD) enzymes capable of citrullinating extracellular proteins in the rheumatic joint, contributing to renewed ACPA generation. This review article focusses on the central function of citrullination, a post‐translational modification of arginine residues in RA. The discussion includes ACPA and related autoantibodies, somatic hypermutation‐mediated escape from negative selection by autoreactive B cells, promotion of the dominance of citrullinated antigens by genetic and lifestyle susceptibility factors and the vicious circle between ACPA‐producing pathogenic B cells and NET‐producing neutrophils in RA.

## Graphical abstract

This article reviews insights into cross‐talk between B cells and neutrophils in seropositive rheumatoid arthritis and its pathogenesis.

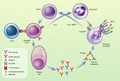

**Abbreviations**
ACPA: anti‐citrullinated protein antibodies
BAFF: B‐cell‐activating factor
CAIA: collagen antibody‐induced arthritis
CarP: carbamylated peptide
CCP: cyclic citrullinated peptide
CIA: collagen‐induced arthritis
MPO: myeloperoxidase
NE: neutrophil elastase
NET: neutrophil extracellular trap
PAD: peptidylarginine deiminase
PTPN22: protein tyrosine phosphatase nonreceptor 22
RA: rheumatoid arthritis
RF: rheumatoid factor
SE: shared epitope
SF: synovial fluid
SHM: somatic hypermutation
SNP: single nucleotide polymorphism

## RHEUMATOID ARTHRITIS

Rheumatoid arthritis (RA) is a chronic, systemic autoimmune disease (reviewed in Ref. [1]). This most common form of inflammatory arthritis affects ~1% of the population worldwide and is more prevalent in women than men. RA is a disabling condition characterized by symmetrical inflammation of synovial joints, with small, peripheral joints most commonly affected. The synovial fluid (SF) becomes enriched in leucocytes and cytokines, and the inflamed synovial membrane develops into an inflammatory pannus, an abnormal layer of blood vessel‐containing tissue which invades the space between the bones, covering bones and cartilage. Unless treated, RA erodes the joint cartilage and bone, causing chronic pain, stiffness, progressive loss of function, disability and, once fusion of bones has occurred, lasting deformities. Up to 40% of patients develop extraarticular RA, which ranges from systemic features, such as vasculitis, to affecting individual organs, for example the lung (e.g., interstitial lung disease) or heart (e.g., pericarditis). With the advent of improved and increasingly sophisticated disease‐modifying anti‐rheumatic drugs, RA has become more manageable in recent years. Although it remains incurable, a combination of early intervention, control of inflammation and prevention of joint damage can culminate in reaching a sustained state of remission.

Rheumatoid arthritis is sometimes regarded not as a single disease but a group of related diseases. Although the pathogenesis of RA is complex and remains incompletely understood, it is clear that this is a long, stepwise process which involves the dysregulation of many cell types, all of which make contributions to this disease. Despite their important contributions, cell types, including T cells, osteoclasts, macrophages and fibroblast‐like synoviocytes, are not discussed here. Instead, this review focusses on the interplay of B cells and neutrophils in autoantibody‐driven (seropositive) RA. Following on from the introduction to these two important cell types, and their critical role in the formation of autoantibodies and protein citrullination, which drive RA, we will discuss risk factors and how they link into RA pathogenesis by promoting citrullination and autoantibody formation.

## B CELLS

Anti‐citrullinated protein antibodies (ACPA; see below for a detailed discussion) are present in serum of >80% of patients with established RA and in ~50% of those with early RA. These autoantibodies can present as much as a decade prior to the onset of any clinical disease [2, 3, 4 indicative of an early loss of tolerance that initiates disease pathogenesis. During B‐cell development, the antibody repertoire is developed. Tolerance is regulated at the central and peripheral checkpoints, when autoreactive B cells are eliminated, become anergic or undergo B‐cell receptor editing [5]. These processes are, however, less strict than those applying to T cells, and some autoreactive B cells escape. Survival of autoreactive B cells may be aided by their genetic predisposition (see below), and self‐reactive low‐affinity antibodies may be masked on anergic B cells. Moreover, somatic hypermutation (SHM) leading to N‐linked glycosylation of the variable region may permit escape from negative selection [6]. Indeed, glycosylation of the ACPA Fc changes during the transition from pre‐arthritis to arthritis with the appearance of a more pro‐inflammatory glycoform of these autoantibodies [7, 8. In the context of collagen‐induced arthritis (CIA) in the mouse, differential glycosylation of ACPA was shown to affect their pathogenicity [9], with IL‐23 and Th17 cells having key roles in promoting pathogenicity [10].

**FIGURE 1:**
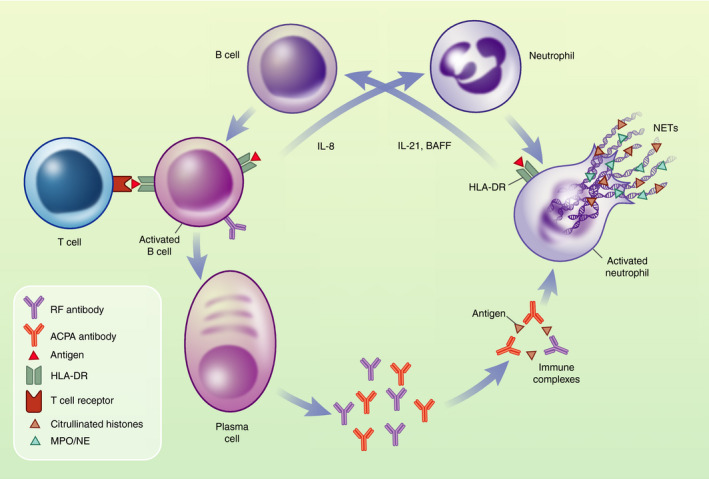
B cells and neutrophils form a vicious circle in RA. Activated B cells release cytokines to crosstalk with other immune cells, with B‐cell‐derived IL‐8 recruiting neutrophils to the synovium [15]. B cells receive T‐cell help with class switching and somatic hypermutation, promoting the development of autoantibodies in a HLA‐DR SE‐dependent fashion. Local plasma cells produce large amounts of autoantibodies including RF and ACPA; these form ICs which activate the complement pathway and promote inflammation, for example by stimulating neutrophils both in the circulation and also in the synovium. Amongst other events, this results in the release of NETs, which are particularly abundant in RA. Myeloperoxidase (MPO), neutrophil elastase (NE) and citrullinated histones are amongst the proteins that decorate NETs [22, 141. Citrullinated histones are thought to act as a continuous source of fresh antigen to B cells, promoting the production of new IgM ACPA. In the synovium, this is promoted by HLA‐DR expressing, activated neutrophils which release cytokines including BAFF and IL‐21, activating B cells [38, 39, 40, 41. In the interest of clarity, RF and ICs are simplified in this cartoon drawing

In addition to pathological autoantibodies, the success of B‐cell depletion therapies targeting CD20 and BAFF [11, 12, 13, 14 identified a critical function of B cells in promoting chronic inflammation in RA. The curious observation that good responsiveness to B‐cell depletion therapy did not correlate with reduced ACPA titres, prompted studies into B cells as drivers of chronic inflammation. Such studies identified continuous antigen‐driven B‐cell activation, proliferating ACPA‐positive memory B cells in both circulation and SF, as well as the production of pro‐inflammatory mediators, notably the neutrophil chemokine IL‐8 by synovial ACPA^+^ B cells [15], suggestive of crosstalk between pathogenic B cells and neutrophils (Figure 1).

## NEUTROPHILS

Neutrophils, the most abundant circulating leucocyte in humans, play a key role in host defence in killing bacteria and fungi either intracellularly, following phagocytosis, or extracellularly [16, 17. Unstimulated neutrophils circulate for only up to 1 day before homing back to the bone marrow where they undergo apoptosis to be cleared by resident macrophages in an anti‐inflammatory process termed efferocytosis. In contrast, upon activation, the short‐lived neutrophil leaves the blood stream and travels to inflammatory sites, such as the inflamed synovium, following gradients of chemo‐attractants and chemokines [17, 18.

Neutrophils are armed with granules loaded with powerful proteases and highly toxic antimicrobial peptides and possess the ability to generate cytotoxic reactive oxygen species (ROS), which can be released into a phagosome or to the outside of the cell. Neutrophils can moreover release their chromatin as ‘neutrophil extracellular traps’ (NETs; see also below), strands of granule protein‐decorated chromatin that have important functions in host defence and that are highly inflammatory [16, 17. Neutrophilic inflammation can be triggered by microbes and sterile stimuli and can impart serious host tissue injury. Immune complexes (ICs) are powerful pro‐inflammatory stimuli of neutrophils that ligate Fc receptors, and trigger effector functions, including, ROS production, degranulation, NETs, chemokine and cytokine generation [19, 20, 21, 22, all of which are thought to contribute to tissue damage incurred in RA. ICs are key to RA. Depending on their ratio of antibody and antigen, ICs can be soluble or insoluble. Both types are abundant in the SF, with further ICs precipitated onto synovial surfaces.

The SF in RA is characteristically sterile, though containing chemokines and cytokines and infiltrated by a large number of leucocytes (>5000/μl), the majority of which are neutrophils [23, 24. At early stages of (clinical) disease, neutrophils are also recruited into the synovial tissue [25, 26, providing indirect clues about the important role of neutrophils in RA.

Circulating neutrophils from RA patients are characterized by an activated phenotype that is characterized by increased ROS, cytokine, protease and NET production as well as delayed apoptosis [27, 28, 29, 30. Intriguingly, patients with RA, as well as rats in an RA model, were found to harbour circulating low‐density granulocytes, with differential cell surface marker and gene expression signatures that are the subject of ongoing investigation [31, 32, 33. SF neutrophils were reported to be more activated still and having a differential gene expression signature compared to circulating cells from the same patient [34]. SF neutrophils are longer‐lived than circulating neutrophils [35, 36, secrete proteases, and release cytokines and chemokines to activate and recruit further neutrophils [29, 37. SF neutrophils also crosstalk with adaptive immune cells, for example by production of B‐cell activating factor (BAFF), inducing B‐cell proliferation and directly contributing to autoantibody production [38, 39. SF neutrophils moreover acquire the ability to present antigen in an MHC‐II‐dependent fashion and drive CD4^+^ T‐cell proliferation [40, 41. This is in keeping with observations that the SF containing neutrophil‐derived cytotoxic products, NETs, cytokines and chemokines produced by neutrophils further inflammation [36, 42, 43.

While experiments with laboratory animals and disease models need to be interpreted with caution, mouse models of RA suggest that neutrophils play a key role in this disease. Antibody‐mediated neutrophil depletion abolished development of K/BxN serum transfer arthritis and also of collagen antibody‐induced arthritis (CAIA) [44, 45, and neutrophil depletion after disease onset resulted in steep decline of CIA [46]. In a series of extensive and elegant investigations, the recruitment of neutrophils to the rheumatic joint in the K/BxN serum transfer model was shown to depend on FcγR as well as a cascade of chemokines, cytokines and chemo‐attractants generated by a variety of cells and their respective receptors on neutrophils [47, 48, 49, 50. Meanwhile, IC‐mediated neutrophil gene expression, including that of pro‐inflammatory mediators, is dependent on CARD9‐dependent regulation of the NFκB pathway downstream of FcγR, Src family kinases and Syk [51, 52, 53. Elegant, very recent work with FcγR‐humanized mouse neutrophils revealed that internalization of ICs permits these neutrophils to become antigen‐presenting cells in a FcγR‐dependent fashion [54]. The resulting neutrophils combined dendritic cell (antigen presentation, T‐cell activation, cytokine production) and neutrophil functions (ROS production, phagocytosis) [54].

## NETS

Neutrophil extracellular traps are web‐like structures that are released by neutrophils. NETs consist of decondensed chromatin decorated with cytotoxic proteins, citrullinated histones, granule‐derived proteins including neutrophilic proteases, myeloperoxidase (MPO), and antimicrobial peptides. Initially described as a pro‐inflammatory cell death mechanism (NETosis), vital NET release was since also demonstrated [55], with the pathway employed being stimulus‐dependent and varying in its NADPH oxidase dependency as well as cleavage of N‐terminal histone tails [56]. Apart from their crucial function in host immunity, trapping and killing pathogens, NETs have important functions in autoimmune diseases including RA [57, 58. Elevated levels of NETs were identified in RA serum and SF [56, 59, 60.

**FIGURE 2:**
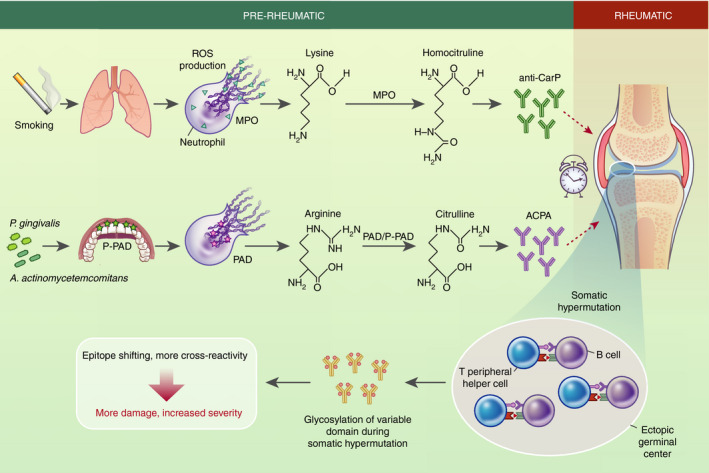
Citrullination and homocitrullination underpin the pathogenesis of RA. Pathogenic autoantibodies including ACPA and anti‐CarP in pre‐rheumatic patients precede the onset of clinical symptoms in RA by up to a decade. In addition to a genetic disposition, triggers at mucosal sites are thought to play a key role in these early events. Smoking‐induced lung inflammation can promote neutrophil‐derived NETs in the lung form and exteriorization of myeloperoxidase (MPO). Together with smoke‐derived cyanate, homocitrullination (also known as carbamylation) of lysine causes the generation of homocitrulline, indirectly promoting the generation of anti‐carP antibodies [86]. Alternative scenarios involve microbial dysbiosis at mucosal surfaces, such as the gingival tissues. Two causative pathogens in gingivitis, *P*. *gingivalis* and *A*. *actinomycetemcomitans* can promote citrullination of antigens by employing bacterial P‐PAD and neutrophil PAD4, respectively [95, 100. Citrullination is a process by which arginine is enzymatically converted to citrulline, generating a highly immunogenic antigen [62]. The events leading to the onset of symptomatic disease years later remain obscure, with possibilities including infection and/or local trauma. In the rheumatic phase, autoantibody maturation occurs locally in the inflamed synovial tissue. B‐cell SHM involving crosstalk of pathogenic B cells and pathogenic peripheral T helper cells occurs in ectopic germinal centres [63]. Strikingly, SHM results in characteristic glycosylation of the antibodies variable domain. This unusual form of SHM does not result in affinity maturation but instead drives epitope shifting and cross‐reactivity of ACPA [110, 115, ultimately resulting in more tissue damage and increased disease severity

Peptidyl‐arginine deiminase 4 (PAD4), a leucocyte‐restricted nuclear PAD, mediates histone citrullination, a post‐translational modification of arginine residues during NET formation (Figure 2). NETs represent an important source of citrullinated (and homocitrullinated) epitopes in RA. Indeed, NET‐associated citrullinated histones represent a continuous source of antigen for B cells and promote the localized generation of ACPA (see below for a detailed section on ACPA) that are able to cross‐react with citrullinated histones in ectopic germinal centres in the inflamed synovial joint [59, 61, 62. Plasma cell differentiation and antibody production are promoted in situ by pathogenic, IL‐21‐producing T peripheral helper cells [63]. Not only are ACPA pathogenic by themselves. Potentially aided by RF (see below for a detailed section on RF), which has the capacity to bind several IgG molecules, ACPA form ICs. These ICs fix complement, accumulate in SF and/or deposit on synovial surfaces to amplify inflammation [64, 65, 66. ICs are powerful stimuli of NET release (e.g., [22, 67, 68). In addition, NETs stimulate neutrophils to produce further NETs and to secrete IL‐8 and BAFF [69], promoting the vicious circle between B cells and neutrophils (Figure 1).

Having introduced neutrophils and B cells, and how they activate one another in established disease, the next section of this review article will discuss genetic and lifestyle factors that predispose to RA. The focus lies on how these factors promote post‐translational modification such as citrullination and/or the NET generation by neutrophils, in turn promoting B‐cell generation of autoantibodies, in particular ACPA, promoting RA pathogenesis.

## GENETIC FACTORS

While it is still unclear why some people ultimately develop RA, there are well‐documented genetic and environmental risk factors. The most significant genetic risk factor identified to date is the class II major histocompatibility locus, with so‐called shared epitope (SE) containing alleles increasing the risk of developing seropositive RA according to epidemiological studies [70, 71. SE sequences ^70^QKRAA^74^, ^70^QRRAA^74^ or ^70^RRRAA^74^ within HLA‐DRB1 are involved in shaping the peptide‐binding pocket of the HLA molecule. The presence of two positively charged residue (lysine, arginine) in residues 71–73 increases the binding capacity of citrullinated peptides over native peptides [72, 73. In addition, citrullinated peptides were also shown to display enhanced binding to HLA‐DQ [74]. Altogether, these mechanisms achieve that citrullinated peptides are preferentially presented, and activate CD4^+^ T‐cell responses, which in turn promote the immune response by helping ACPA‐producing B‐cell antibody maturation (class switching and somatic hypermutation).

Additional genetic risk factors have been attributed to single nucleotide polymorphisms (SNPs) in a range of genes. The most prominent of these is a SNP, C1858T, in the leucocyte‐restricted protein tyrosine phosphatase nonreceptor 22 (PTPN22) which encodes the R620W variant [75]. This allele and especially C1858T homozygosity cause an elevated risk of developing RA, earlier disease onset and more aggressive disease, with RF positivity conferring increased odds. R620W PTPN22 was reported to drive blunted BCR signalling and reduced B‐cell apoptosis, resulting in increased escape of poly‐ and autoreactive B cells from central and peripheral tolerance in humans and mouse models [76, 77, 78. PTPN22 is most highly expressed in neutrophils, with R620W reported to promote transendothelial migration and ROS production in human neutrophils [79], while in a mouse model *Ptpn22* deficiency caused decreased pro‐inflammatory responses to IC stimulation without affecting neutrophil recruitment to inflammatory sites [80]. PTPN22 was moreover shown to physically interact with and inhibit PAD4 in a phosphatase‐independent fashion. Indeed, R620W PTPN22 promoted enhanced citrullination which resulted in increased NET production by neutrophils and in defective Th2 and Th17 cytokine production by peripheral blood‐derived mononuclear cells [81, 82.

## LIFESTYLE: SMOKING

The major environmental factor associated with developing RA is smoking, which in combination with the HLA‐DR SE confers a significantly increased susceptibility to ACPA‐positive RA according to epidemiological studies (e.g., [83, 84). This suggests that smoking may provide an external trigger for those already carrying genetic risk factors to develop RA, and identifies the lung as an important mucosal site in RA development (Figure 2). Indeed smokers' lung biopsies were characterized by upregulated PAD2/4 expression and increased protein citrullination [85, 86. Recently, enhanced carbamylation and anti‐CarP (see below) were also found in smokers and smoke‐exposed laboratory mice [87, 88, directly implying neutrophils. Interestingly, neutrophils isolated from smokers were moreover shown to be more prone to NET production in a nicotine‐dependent fashion. In experimental mice, too, nicotine promoted NET production and caused more severe disease scores in CIA [89, 90.

## DYSBIOSIS: EXAMPLE PERIODONTITIS

Links between the disruption of the beneficial relationship between commensal bacteria and the host at mucosal surfaces were documented in inflammation, with dysbiosis of the oral microbiota and periodontitis most clearly associated with RA pathogenesis [91] (Figure 2). A statistically significant association between RA and periodontitis was shown in a number of clinical studies, with RA patients with severe periodontitis suffering from more severe RA [92]. Associations between ACPA and severity of periodontitis in RA and pre‐RA patients were also described [93, 94. A possible explanation for this observation rests with two key periodontal pathogens. *Porphyromonas gingivalis* expresses a bacterial deiminase, PPAD, which citrullinates C‐terminal arginines of bacterial and host origin in a calcium‐independent fashion, potentially contributing to the breaking of tolerance [95]. In the context of CIA in the mouse, *P*. *gingivalis* PPAD activity could increase inflammatory arthritis [96, 97. Notably, *P*. *gingivalis* also affects the cytokine response of gingival epithelial cells, driving recruitment of Th17 cells and neutrophils via CCL20 and CXCL8. *P*. *gingivalis* was further shown to trigger release of non‐bactericidal NET production by neutrophils, encouraging microbial growth and increasing citrullinated antigens in the periodontal space [98, 99. In a separate mechanism, *Aggregatibacter actinomycetemcomitans* was shown to use its pore‐forming leucotoxins to induce hypercitrullination and NET formation by neutrophils, which in turn increased ACPA in the periodontal space [100].

## AUTOANTIBODIES

As laid out above, the earliest and perhaps most conspicuous feature of RA are autoantibodies, which can be present years or even decades prior to onset of clinical symptoms (pre‐RA), with epitope spreading and expansion of autoantibodies occurring prior to the onset of clinical disease.

### Rheumatoid factor

Rheumatoid factor refers to antibodies directed against the Fc region of IgG and was described in the 1940s. Despite its high prevalence in RA (up to 80% of patients) and positive association with more severe disease progression, RF is not restricted to RA, making it an unreliable diagnostic marker (reviewed in Ref. [1]). RF can undergo class switching, with IgM and IgA RF most commonly observed in RA. RF moreover undergoes somatic hypermutation and affinity maturation in RA. By recognizing IgG, RF is perfectly suited to forming large ICs, and to promoting deposition of complement to improve clearance of excess antibodies, the likely function of natural RF [101]. However, as laid out above, ICs also play a key role in promoting persistent inflammation including via neutrophils.

### Anti‐citrullinated protein antibodies

Anti‐citrullinated protein antibodies represent a second class of highly prevalent autoantibodies found in RA. ACPAs are present in the serum of 80–90% of patients with established RA and in up to 20% of their first‐degree relatives [102]. For diagnostic purposes, presence of ACPA in the serum is detected by using cyclic citrullinated peptide (CCP2/CCP3) assays, where synthetic CCPs are used that were optimized for optimal ACPA capture. Serum ACPA react with peptides derived from a range of citrullinated protein antigens that are found in the rheumatic joints, including fibrin vimentin, α‐enolase and histones [62, 103, 104, 105, 106, with a high degree of cross‐reactivity between substrates observed for individual monoclonal ACPAs [107]. Interestingly, it was recently suggested that improved screening might detect autoantibodies in seronegative RA patients that do not cross‐react well with the CCPs used in current clinical testing [108]. Although the presence of ACPA in a person without clinical symptoms does not predict that they will be developing RA, contrasting with RF, the presence of ACPA is highly specific to RA, making them a useful diagnostic tool. ACPA is moreover indicative of more severe disease progression [109]. In RA, ACPA associate with RF and the HLA SE, as laid out above.

Anti‐citrullinated protein antibodies undergo class switching, extensive somatic hypermutation as well as conspicuous variable region glycosylation and epitope spreading, leading to cross‐reactive antibodies (Figure 2). However, affinity maturation is limited and even serum IgG ACPA is characterized by low binding affinity for citrullinated protein [110, 111, 112, 113, 114, 115. Interestingly, despite the short half‐life of 5.9 days of IgM in RA [116], and the fact that long‐lived IgM‐secreting plasma cells are not described in humans, IgM ACPA continues to be present in the serum of RA patients, or can occur at later stages in previously IgM ACPA‐negative patients [117, 118. This suggests that new citrullinated antigen‐specific B cells continuously generate new ACPA, implying the continued generation of fresh citrullinated antigens, for example due to neutrophil‐mediated production of NETs [57, 59, 60.

### Anti‐CarP

Processes related to citrullination also lead to secondary modification of protein epitopes that are similarly antigenic. Carbamylation, also known as homocitrullination, is the post‐translational modification of lysine to homocitrulline (Figure 2). Unlike citrullination, carbamylation is a chemical modification that does not rely on an enzyme, but occurs on the presence of cyanate as a myeloperoxidase‐dependent oxidation of thiocyanate which is abundant in smokers [86]. Carbamylated peptides were predicted to bind SE alleles [119]. Anti‐CarP (carbamylated peptide) antibodies are directed against carbamylated proteins and frequently cross‐react with citrullinated proteins [119]. Anti‐CarP are present in 45% of RA patients including some of those who are ACPA‐negative. Like ACPA, anti‐CarP antibodies are very specific to RA, present in the serum up to 10 years before the onset of clinical symptoms, and are associated with NETs [58, 120.

### Anti‐PAD antibodies

Neutrophils express three PAD enzymes, PAD2/3/4. Extracellular, active PAD was observed in cell‐free SF of RA patients [121]. Neutrophil NETosis was found to result in release of free PAD2/4 in vitro, raising the possibility that dying neutrophils may be the source of free PAD2/4 [122]. However, neutrophils were also found to spontaneously secrete or expose PAD2/4 [123], providing an alternative explanation for the citrullination of extracellular targets in the rheumatic joint. Interestingly, PADs themselves also serve as RA autoantigens. Indeed, ~30% of RA patient, but not control sera, were found to be anti‐PAD4 positive, with anti‐PAD4 positivity occurring prior to (clinical) disease onset and being a marker of severe disease [124, 125, 126. PAD4 requires >100 μM Ca^2+^ to display any catalytic activity in vitro and mM Ca^2+^ concentrations for optimal activity. This vastly exceeds the Ca^2+^ concentration of the resting cell [127], but can be achieved following activation and opening of Ca^2+^ channels [128]. The fact that histone citrullination occurs physiologically to regulate gene expression [129] moreover suggests the existence of additional mechanisms that allow PAD enzymes to be active at lower Ca^2+^. Fascinatingly, a subset of cross‐reactive anti‐PAD3/4 antibodies were shown to inducing a conformational change into PAD4 when binding to it, rendering it hyperactive by which vastly reduced its Ca^2+^ requirement to a physiological level [130, 131. Presence of these truly pathogenic anti‐PAD3/4 antibodies correlates with particularly aggressive disease.

## CONCLUDING REMARKS

Despite decades of research into RA, the trigger that precipitates the original break of tolerance and the nature of additional events that initiate clinical disease remain obscure. It is clear, however, that a combination of genetic predisposition, lifestyle choices and dysbiosis can all contribute to culminate in clinical disease. Citrullination, much of it likely to be neutrophil‐derived, and ACPA lie at the heart of the initial break of tolerance, and subsequently, a vicious circle between neutrophils and B‐cell‐derived autoantibodies/ICs plays out. Neutrophils are often portrayed as uniquely pro‐inflammatory; however, this is likely not the whole truth. Not only do neutrophils clear insoluble ICs from biological fluids, thereby reducing these highly pro‐inflammatory stimuli, but the same ICs also induce neutrophil apoptosis [132, 133, 134, a cell death that promotes the resolution of inflammation [135]. A separate intriguing notion is moreover that physiologically citrullination may occur in order to limit excessive inflammation. Free histones are extremely cytotoxic [136]; however, their citrullination in NETs renders them more susceptible to proteolytic degradation. It also reduces their ability to stimulate further NET production [137, 138. In a similar way, citrullination may confer the host with a degree of protection from the highly toxic antibacterial peptide LL37 [139, 140.

## CONFLICT OF INTEREST

The authors declare no competing interests.

## AUTHOR CONTRIBUTION

UK and SV wrote the manuscript and designed the figures.
